# Immune Memory After Respiratory Infection With *Streptococcus pneumoniae* Is Revealed by *in vitro* Stimulation of Murine Splenocytes With Inactivated Pneumococcal Whole Cells: Evidence of Early Recall Responses by Transcriptomic Analysis

**DOI:** 10.3389/fcimb.2022.869763

**Published:** 2022-06-20

**Authors:** Isabelle Franco Moscardini, Francesco Santoro, Monica Carraro, Alice Gerlini, Fabio Fiorino, Chiara Germoni, Samaneh Gholami, Elena Pettini, Donata Medaglini, Francesco Iannelli, Gianni Pozzi

**Affiliations:** ^1^ Microbiotec srl, Siena, Italy; ^2^ Laboratory of Molecular Microbiology and Biotechnology (LAMMB), Department of Medical Biotechnologies, University of Siena, Siena, Italy

**Keywords:** *Streptococcus pneumoniae*, *in vitro* stimulation, Transcriptomic Analysis, Recall immune responses, lung infection, cytokines/chemokines

## Abstract

The *in vitro* stimulation of immune system cells with live or killed bacteria is essential for understanding the host response to pathogens. In the present study, we propose a model combining transcriptomic and cytokine assays on murine splenocytes to describe the immune recall in the days following pneumococcal lung infection. Mice were sacrificed at days 1, 2, 4, and 7 after *Streptococcus pneumoniae* (TIGR4 serotype 4) intranasal infection and splenocytes were cultured in the presence or absence of the same inactivated bacterial strain to access the transcriptomic and cytokine profiles. The stimulation of splenocytes from infected mice led to a higher number of differentially expressed genes than the infection or stimulation alone, resulting in the enrichment of 40 unique blood transcription modules, including many pathways related to adaptive immunity and cytokines. Together with transcriptomic data, cytokines levels suggested the presence of a recall immune response promoting both innate and adaptive immunity, stronger from the fourth day after infection. Dimensionality reduction and feature selection identified key variables of this recall response and the genes associated with the increase in cytokine concentrations. This model could study the immune responses involved in pneumococcal infection and possibly monitor vaccine immune response and experimental therapies efficacy in future studies.

## 1 Introduction

*Streptococcus pneumoniae* is a major human pathogen responsible for various diseases, including life-threatening conditions such as pneumonia, sepsis, and meningitis (Weiser et al., 2018). Current vaccines have been very efficient in reducing the death toll caused by this pathogen. However, strains not covered by the available vaccines represent a growing concern, demanding new serotype-independent strategies (Kaplan et al., 2013; Pichichero, 2017; Briles et al., 2019). Models to assess the response to new vaccine candidates would be of great use.

*In vitro* stimulation with live or killed bacteria has been used for decades for understanding the host’s response to different pathogens, including *S. pneumoniae* (Zhan and Cheers, 1995; Schultz et al., 1998; Wu et al., 2011; de Stoppelaar et al., 2016). This technique has also been applied in vaccine studies, characterizing the immune response after a second stimulus (Paranavitana et al., 2010; Moffitt et al., 2011; Shao et al., 2015). Changes in gene expression and in cytokine concentration were metrics assessed by some of these works to study the immune profile of pneumococcal infection. In the present work, we propose the combination of transcriptomic and cytokine assays from murine splenocytes to assess the immune memory built in the days following pneumococcal infection.

The spleen plays a vital role in host defenses against encapsulated blood-borne pathogens due to its elevated perfusion and efficient immune surveillance of the circulatory system (Cerutti et al., 2013). In a pneumococcal bacteremia model, bacteria present a tropism to the spleen, and macrophages present in the splenic Red Pulp (RP) are responsible for an initial binding and subsequent clearance of *S. pneumoniae* mediated by mature neutrophils present on the RP (Deniset et al., 2017; Ercoli et al., 2018). Moreover, the splenic Marginal Zone (MZ) is a crucial area of antigen presentation to MZ B cells that are capable of rapidly differentiating into plasmablasts, secreting low-affinity IgM and IgG (Cerutti et al., 2013).

RNA sequencing technologies permit us to gain insights into the host’s response due to the possibility of analyzing the changes in gene expression in different conditions. The transcriptomic information can be integrated with other biological layers or clinical data, permitting a more comprehensive understanding of biological processes in response to perturbations such as infection and vaccination.

In the current work, we propose the study of the host systemic responses to a pneumococcal lung infection by assessing gene expression and cytokine profiles of splenocytes, identifying biological pathways and the key features involved in this process.

## 2 Methods

### 2.1 Animals and Animal Infection

Seven-week female C57BL/6 mice (Charles River Italia, Italy) were treated according to national guidelines (Decreto Legislativo 26/2014) utilizing the three R’s principles. Animals were maintained under specific pathogen-free conditions in the animal facility of the Laboratory of Molecular Microbiology and Biotechnology (LA.M.M.B.), Department of Medical Biotechnologies at University of Siena, at 20-24°C, with 55 ± 10% of humidity, with food and water *ad libitum*. The study was approved by the Italian Ministry of Health with authorization n° 304/2018-PR. As previously described (Kadioglu et al., 2011) male and female mice respond differently to pneumococcal infection and, therefore the use of only female mice can be considered a limitation of this study.

**Figure 1:**
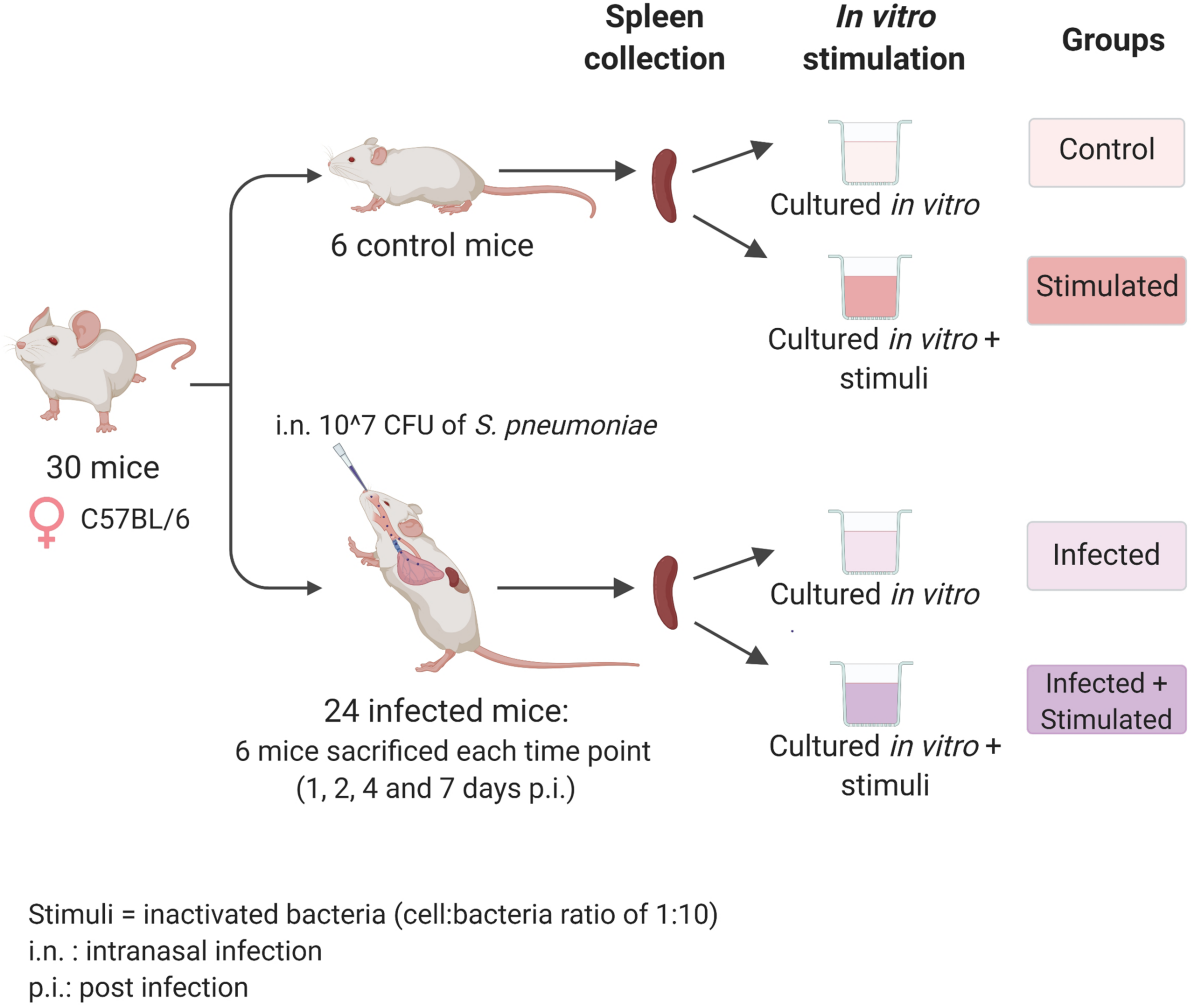
Experimental design. Seven-weeks old C57BL/6 female mice were infected intranasally with a dose of 10^7 CFU/mouse of pneumococcal serotype 4, TIGR4 strain. After 1, 2, 4, and 7 days post-infection each group of mice (n=6) was euthanized and their spleens were collected for the isolation of splenocytes. Splenocytes from each single mouse were stimulated with formalin-inactivated TIGR4 (cell:bacteria 1:10), or maintained in cRPMI medium alone. The incubation was performed at 37°C in 5% CO_2_ in a period of 6 hours for samples used in the RNA-sequencing experiment or 72 hours for samples used in the Bio-Plex Multiplex immunoassay. Non-infected mice were euthanized at day 0, splenocytes were collected and maintained in the same *in vitro* conditions of the unstimulated group and used as controls. Created with biorender.com.

Mouse-passaged TIGR4 strain of *S. pneumoniae* (Gerlini et al., 2014) was inoculated 1:50 in TSB (Tryptic-Soy Broth, Becton Dickinson, USA) supplemented with 0.1% of glucose (PanReac, Applichem, Italy), 1% of yeast extract (Oxoid, UK) and 0.016 M K_2_HPO_4_ (Sigma-Aldrich, USA) (TSB-GYP). The bacterial culture in mid-exponential phase (≈OD_590 =_ 0.6), was centrifuged at 2,000 x *g* for 10 minutes and resuspended in an appropriate volume of saline. Before the centrifugation, the culture was Gram-stained and bacterial vital counts were performed using the multilayer plating method (Iannelli et al., 2021). Each mouse was anesthetized by intraperitoneal administration of 15 mg/kg tiletamine hydrochloride/zolazepam and 4 mg/kg xylazine and intranasally infected by instillation of 10^7 CFU of TIGR4, prepared as described above, in the volume of 25 μl/nostril in TSB. Mice were euthanized at different time points with overdose of anesthesia and cervical dislocation, as shown in **Figure 1**. Non-infected mice composed the baseline group. Each group included 6 animals.

### 2.2 Sample Collection

After aseptic removal at different time points (days 0, 1, 2, 4, and 7 after infection), spleens were meshed onto 70 µm nylon screens (Sefar Italia, Italy) using a scalpel and scraper. Cells were washed two times in RPMI (Sigma-Aldrich) supplemented with 10% of fetal bovine serum (FBS, Gibco, USA) and 1% of penicillin-streptomycin (Sigma-Aldrich)(cRPMI), treated with red blood cells lysis buffer according to manufacturer instruction (eBioscience, USA), and resuspended in cRPMI for cell counting by an automatic cell counter (Bio-Rad, USA). Lungs were aseptically removed, meshed onto 40 µm nylon screens (Sefar Italia, Italy), suspended in 1 ml of TSB containing glycerol at a final concentration of 10% and frozen at -70°C.

### 2.3 Bacterial Cells Counts in Lungs

Bacterial colony forming units (CFUs) were determined by plating appropriate dilutions of frozen lungs using a multilayer plating procedure (Iannelli et al., 2021). The lower limit of detection was 10 CFU/ml. CFUs were counted at 1, 2, 4, and 7 days after intranasal infection and in the mock infected control group.

### 2.4 Preparation of Inactivated Whole Cells of *S. pneumoniae*

TIGR4 was inoculated 1:1000 (v:v) in 1 L of pre-heated (37°C) TSB-GYP in a GLS80 1 liter bottle (Duran, USA). Temperature was maintained constant at 37°C, the pH was continuously measured with a probe (InPro3030, Mettler Toledo) and kept at 6.9 by peristaltic pump controlled addition of 3M NaOH. Agitation was set at 100 rpm. Growth was monitored by aseptically drawing aliquots and measuring their OD_590_ in a Spectronic 200 spectrophotometer (ThermoFisher). At the peak OD_590_ (about 2.5, corresponding to 10^9 CFU/ml) bacteria were harvested by centrifugation, resuspended in PBS/10% glycerol and frozen at -70°C. TIGR4 bacteria were then thawed and inactivated by treatment with 1.5% formalin for 2 hours on a roller mixer at room temperature, then washed twice and resuspended in water.

### 2.5 Splenocyte Stimulation and Cytokine Secretion Assay

Splenocytes were cultured in a U-bottom 96-well plate in triplicate for 72 hours at 37°C with 5% CO_2_ in cRPMI.

Splenocyte cultures were incubated in the presence or not of inactivated TIGR4, at a cell:bacteria ratio of 1:10. Unstimulated control splenocytes were cultured in cRPMI alone, and positive control splenocytes were stimulated with 50 ng/ml of phorbol 12-myristate 13-acetate (PMA) and 1 µM of Ionomycin (both from Sigma-Aldrich). After stimulation, cells were harvested and centrifuged at 500 x *g* for 15 minutes at 4°C. The supernatant was recovered and frozen at -70°C for subsequent Luminex immunoassay.

A broad screening panel consisting of a biologically-relevant collection of adaptive immunity cytokines, pro-inflammatory cytokines, and anti-inflammatory cytokines was used. In particular, IL-1α, IL-1β, IL-2, IL-3, IL-4, IL-5, IL-6, IL-9, IL-10, IL-12p40, IL-12p70, IL-13, IL-17, G-CSF, GM-CSF, IFNg, and TNF-α, and of the chemokines Eotaxin, KC, MCP-1 (MCAF), MIP-1α, MIP-1β, and RANTES production by *in vitro* stimulated splenocytes was assessed with the BioPlex pro mouse cytokine group 1 - panel 23-plex immunoassay (Bio-Rad, USA) according to manufacturer guidelines, and analyzed by Bio-Plex Magpix Multiplex reader (Bio-Rad). Cytokine and chemokine concentration was expressed as picograms per milliliter (pg/ml) and were calculated using Bio-Plex Manager 6.1.

### 2.6 Splenocyte Stimulation for RNA-Sequencing

In a U-bottom plate, 1 x 10^6 splenocytes/well were seeded in quintuplicate and cultured for 6 hours at 37°C with 5% CO_2_ in cRPMI in the presence of inactivated TIGR4 at a cell:bacteria ratio of 1:10. Unstimulated control splenocytes were cultured in cRPMI alone. Upon stimulation, cell replicates were centrifuged at 500 x *g* for 10 minutes at 4°C. The supernatant was discarded, the pellet resuspended in 50 µl of lysis buffer RA1 (Macherey-Nagel, Germany), flash-frozen in liquid nitrogen, and stored at -70°C for subsequent RNA extraction.

### 2.7 RNA Extraction, Library Preparation, and Sequencing

The RNA purification was performed with the NucleoSpin^®^ RNA kit (Macherey-Nagel) following manufacturer’s instructions, and, before DNAse treatment, the extracted RNA was quantified by the Qubit^®^ 2.0 Fluorometer (Invitrogen by Thermo Fisher Scientific, USA), using the Qubit RNA BR (Broad-Range) Assay Kit.

Contaminating DNA was removed from the extracted RNA by adding 10X TURBO DNase Buffer (TURBO DNase, Ambion by Thermo Fisher Scientific) and 1 µl of TURBO DNase (Ambion), and samples were incubated at 37°C for 30 minutes. After purification by the RNA Clean & Concentrator Kit (Zymo Research, USA), the obtained RNA was quantified using the Qubit^®^ RNA BR Assay Kit.

Library preparation was performed as described in a previous publication (Santoro et al., 2021), using the Ion AmpliSeq™ Transcriptome Mouse Gene Expression Kit from AmpliSeq (Thermo Fisher Scientific), allowing the amplification of 23,930 target genes. Libraries were diluted to 50 pM and pooled in equal volumes (7 μl), with eight individual samples per pool and loaded onto Ion PI™ Chip v3 using the Ion Chef™ Instrument. Sequencing was performed using Ion Proton™ Sequencer.

All described steps were performed according to the manufacturer’s instructions.

### 2.8 RNA-Sequencing Data Analysis

R software in version 3.6.3 was used for transcriptomic data analysis. The DESeq2 package (Love et al., 2014), performs differential expression analysis and multiple test correction, returning values of LogFC, and adjusted p values. Genes with an adjusted p-value smaller than 0.05 and an absolute value of log2 Fold Change greater than 0.5 were classified as differentially expressed and then used in the enrichment analysis performed by the hypergeometric test from the *tmod* package (Weiner 3rd and Domaszewska, 2016) using the Blood Transcription Modules (BTM) database (Li et al., 2014).

### 2.9 Cytokine Data Analysis

R software in version 3.6.3 and the software GraphPad Prism 8.0 were used to perform the statistical analysis. The cytokine concentrations between stimulated and unstimulated samples were compared using the Wilcoxon signed-rank test, a non-parametric test used to compare two related samples. Samples from different time points were analyzed using the Mann-Whitney test, a non-parametric test for non-matched samples. A p-value ≤0.05 was considered statistically significant.

### 2.10 Biomarker Analysis

The DaMiRseq (Chiesa et al., 2018) package was used to find possible biomarkers of the host response to the second stimulus, the inactivated bacteria. Stimulated samples were selected and divided into three groups: baseline, infected samples at days 1 and 2 (early time points), and infected samples at days 4 and 7 (late time points). Following a pipeline that permits normalization, data adjustment, and feature selection, the DaMiRseq package ranked the most important features to distinguish the three classes. The number of selected genes was chosen based on the importance established by the package; genes with a scaled importance score higher than 0.5 were chosen (**Supplementary Image 1**).

### 2.11 Data Integration

To integrate RNA-sequencing results and the Cytokines Bioplex, we selected the concurrent samples from both experiments, and we performed the sparse version of Partial Least Squares (sPLS), provided by the MixOmics package. The PLS is a multivariate method to integrate two high dimensional matrices, maximizing the covariance between components from two data sets, in our case, the transcriptomic and cytokines assay data. The sparse version, sPLS, applies LASSO penalization in each pair of loading vectors from PLS, performing feature selection and providing the correlation values between the main features in each data set (Lê Cao et al., 2008).

According to the Q^2^ criterion, two components would be sufficient to run the model (Q^2^ of 0.33931900 and 0.08639437). As suggested by the *tune*.*spls* function, the optimal variable number was chosen in each component resulting in 16 genes for component 1 and 25 from component 2.

## 3 Results

Groups of six C57BL/6 mice were intranasally inoculated with 10^7 CFUs of TIGR4 *S. pneumoniae* to generate lung infection. To study the systemic response induced at early time points afterinfection, we sacrificed animals after 1, 2, 4, and 7 days andisolated their splenocytes. We then stimulated splenocytesfrom each single mouse with formalin-inactivated whole pneumococcal cells and investigated the host responses by transcriptomic analysis and assessment of cytokine production (**Figure 1**).

### 3.1 Evidence of Pneumococcal Lung Infection in Mice

**Figure 2:**
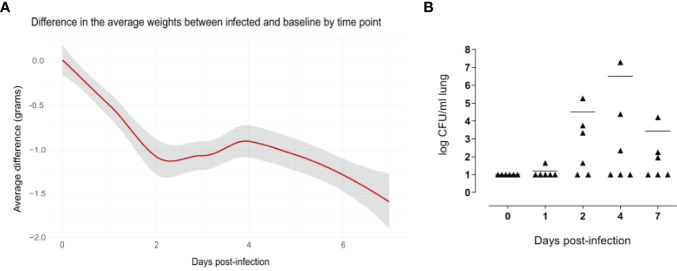
Evidence of pneumococcal lung infection. Comparison of body weight variation of infected versus baseline **(A)**. Infected (n=36) and uninfected (n=5) mice were weighed every 24 hours for a period of 7 days. Values were obtained subtracting the pre-inoculum body weight from the body weight at each time-point, and then subtracting the mean in the control group from mean of the infected group in each time point. The average differences are expressed in grams and the gray shade represents the 0.95 confidence interval. From days 2 to 7 there were significant differences between the infected and baseline groups (p < 0.05, calculated using Mann-Whitney test. Pneumococcal cell counts in the lungs **(B)**. The lungs of mice sacrificed at 1, 2, 4 and 7 days after infection (n=6 per group) were collected, homogenized in a final volume of 1 ml and plated using a multilayer plating procedure. Pneumococcal cells were counted after 24 hours and 48 hours of incubation. Lungs of uninfected mice (0 days post-infection) were plated as a negative control. Data are expressed as CFUs/ml lung. The lower limit of detection was 10 CFU/ml lung. Average cell counts had a peak at day 4. For each time point, there were at least 2 mice without detectable pneumococci.

The weight loss of animals after infection is a critical clinical parameter of disease in the mouse model of infection, evaluated in different challenge murine models with different pathogens (Trammell and Toth, 2011; Pettini et al., 2015; Fiorino et al., 2021). Mouse body weight was measured every 24 hours for a period of seven days. Uninfected mice increased their body weight over time, which reflected their health status. Compared to naive mice, infected mice experienced a significant decrease in body weight soon after infection, and the average difference in the weight between the classes increased over time, being 1.07 grams at day 2 after infection and 1.52 grams at day 7 (**Figure 2A**).

The significance of these findings was assessed using the Mann-Whitney test, which showed significant differences in the weight from day 2 to day 7 after infection, indicating a long-lasting effect of the infection.

Pneumococcal cells were counted in the lungs of infected mice. Cell counts had a peak at day 4 after infection (**Figure 2B**). It is worth to note that, for each time point assayed, 2-5 mice had no detectable pneumococcal cells, suggesting that mice are able to spontaneously clear pneumococcal lung infection at an infectious dose of 1x10^7 CFUs of *S. pneumoniae* TIGR4. When setting up the mouse model, we also counted pneumococcal cells in the blood of six mice at 6 and 12 hours after intranasal infection, and in the blood of 12 mice at 24 and 96 hours after infection. Of those, only one animal had detectable pneumococcal cells in the blood (2.4x10^3 CFU/ml) at 24 hours after infection, suggesting that the infection is essentially limited to the mouse lungs without significant systemic spreading.

### 3.2 *In vitro* Splenocyte Stimulation With Pneumococcal Strain TIGR4 Activates Several Genes Related to Both Branches of the Immune System

Transcriptomic data from spleens of infected and uninfected mice with or without homologous *in vitro* stimulation were analyzed. We performed an Independent Principal Component Analysis (IPCA) and its sparse version, sIPCA, both proposed by MixOmics package (Yao et al., 2012), (i) to observe the distribution of our data, (ii) to understand how stimulation at different time points affects the clustering of samples, (iii) to identify the genes responsible for the main variance among samples, and (iv) to find possible outlier samples.

**Figure 3:**
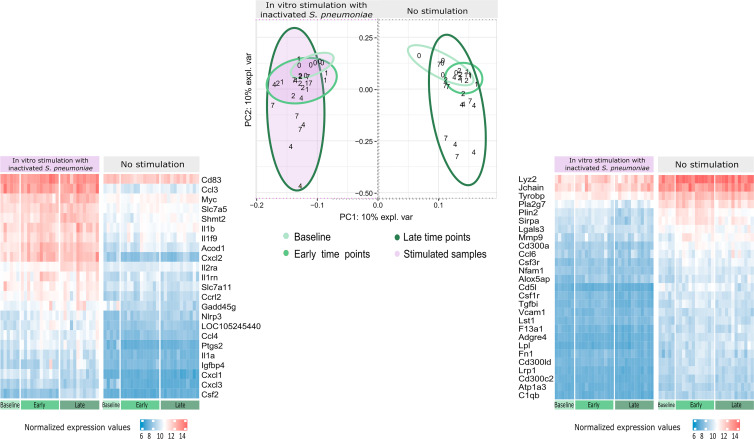
Distribution of gene expression data and changes after stimulation. The sIPCA proposed by MixOmics revealed two main clusters, one formed by stimulated samples (shaded in purple) and other formed by unstimulated samples. The IPC1 (x-axis) represents the variance between stimulated and unstimulated samples of infected and non-infected mice. IPC2 (y-axis) shows the variance between baseline samples with respect to the other groups in different study days (1, 2, 4, 7). After infection, samples from days 1 and 2 (early time points) form a smaller cluster closer to baseline samples (lighter green), while samples from days 4 and 7 (late time points) present a higher dispersion (darker green). The normalized expression values for the 50 most important genes selected by the sIPCA in the first component were represented in heatmaps, with lower values represented in blue and higher values in red and time points represented in the bottom part, following the same green scale of the sIPCA. The heatmap on the right, shows the 27 genes positively correlated with the first component, which include positive and negative regulators of the immune system and drive the formation of the cluster of unstimulated samples. The heatmap on the left shows the 23 genes negatively correlated with the first component, driving the stimulated samples to form a separate cluster. Most of these genes are related to cytokines and chemokines (Csf2, Ccl4, Il1a, Il1b, Il1rn, Il2ra, Cxcl1, Cxcl3, Cxcl2, Ccl3, Slc7a5) while others are related to immunity and inflammation (Cd83, Acod1, Slc7a11, Gadd45g, Nlrp3, Ptgs2 and Igfbp4).

The IPCA approach (**Supplementary Image 2**) yielded a better clusterization among experimental groups and time points compared to PCA (data not shown). An outlier control sample was detected and removed. The sparse version of the IPCA (sIPCA, **Figure 3**) applies soft-thresholding in the independent loading vectors in IPCA, performing feature selection. The graph shows the presence of two well-defined groups in the sIPC1: the stimulated and unstimulated samples.

To better understand the genes that drive the formation of these clusters, the normalized expression values of the 50 genes selected by the first component of the sIPCA were divided into two heatmaps (**Figure 3**). Genes positively correlated with the first component (driving the unstimulated cluster) included positive and negative regulators of the immune response, and they presented a decreased expression after stimulation. The genes negatively regulated with the first component (clustering the stimulated samples) were related to cytokines, chemokines, and inflammation, all of them presenting an increased expression compared to unstimulated samples.

### 3.3 Stimulation of Splenocytes From Infected Mice Highlights Biological Pathways of Pneumococcus Infection

We then proceeded with the differential expression analysis using the *DESeq2* package. To understand the biological alterations caused by the infection and the subsequent *in vitro* stimulation with inactivated pneumococcus, enrichment analysis was performed using three different comparisons. (i) Spleens from infected mice, (ii) stimulated spleens from non-infected mice, and (iii) stimulated spleens from infected mice, at different time points after infection, were all compared with control spleens.

**Figure 4:**
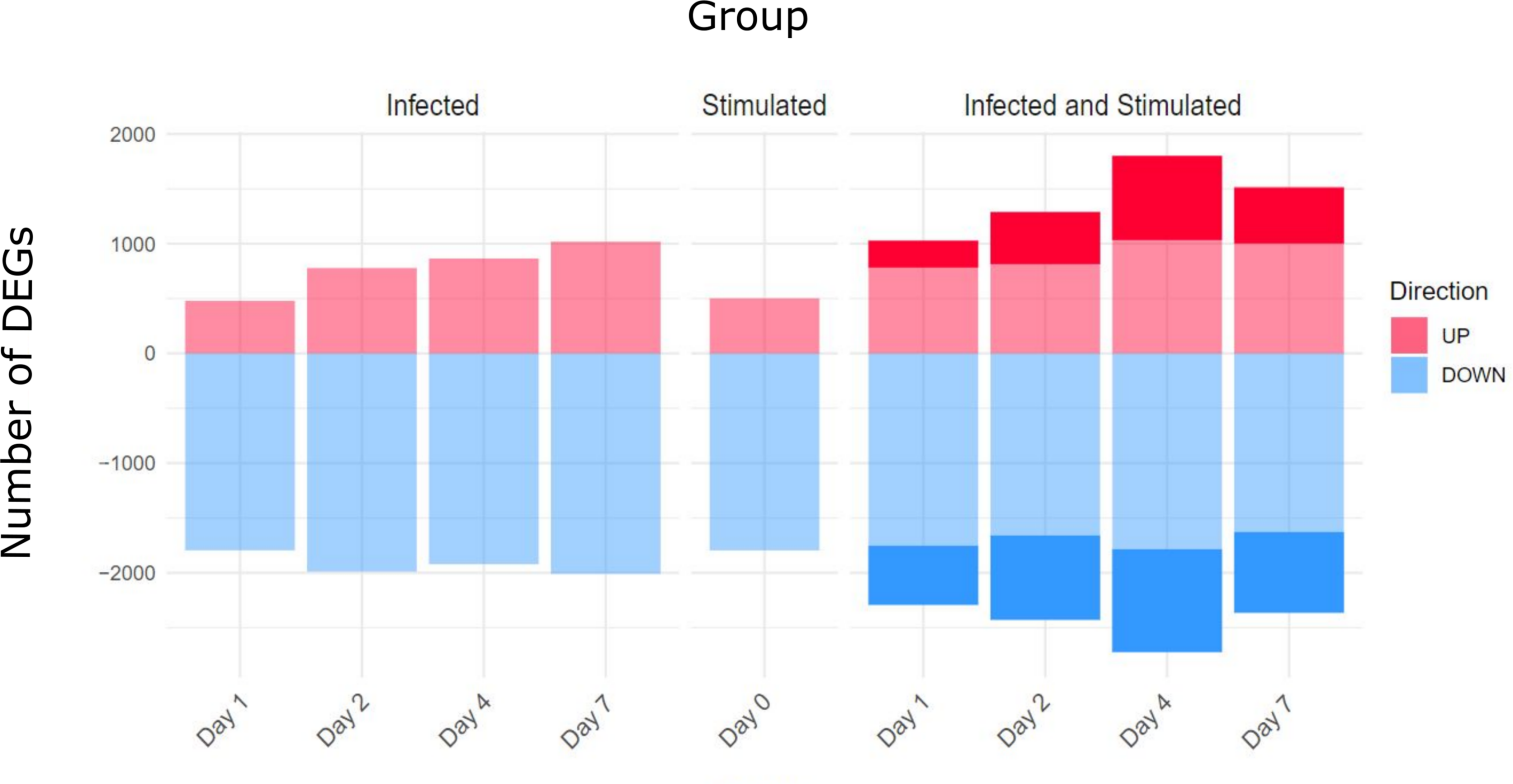
Differentially Expressed genes (DEGs) for comparison and Time point. Three main comparisons against baseline controls were defined: infected samples, stimulated samples and infected and stimulated samples. Differentially Expressed Genes (DEGs) were obtained using the DESeq2 package and establishing thresholds of FDR < 0.05 and absolute logFC > 0.5. The stimulation of spleens from infected mice led to a higher number of DEGs compared to only infected or only stimulated spleens (number of specific genes highlighted in the figure).

The number of differentially expressed genes (DEGs) for each condition at each time point is presented in **Figure 4**. As expected, the stimulation of infected samples led to a higher number of DEGs compared to only infected samples, including specific genes that were not differentially expressed in the infection or stimulation alone. Days 4 and 7 presented the highest values of specific DEGs, in particular, new immune related genes and microRNAs were found, such as Il2, Foxp3, Il16a, Ccr1 and Mir155hg.

**Figure 5:**
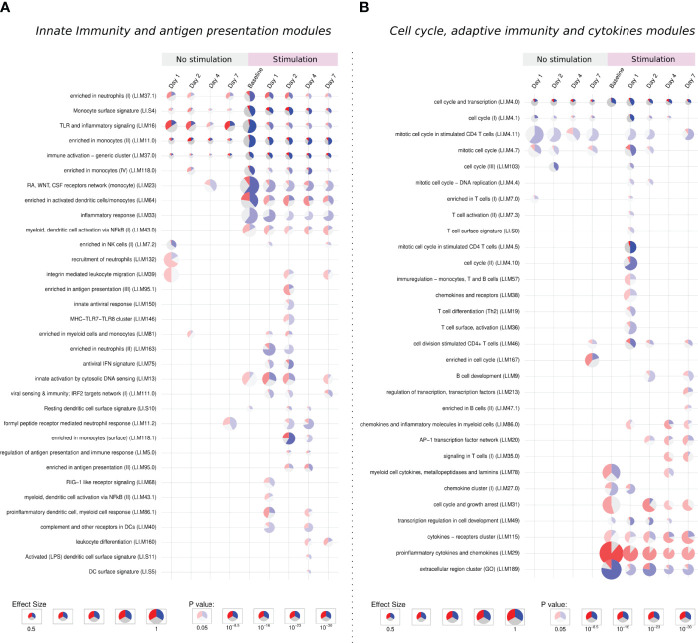
Tmod enrichment analysis. From the list of genes provided by the DESeq2 package, genes were selected by their FDR value (< 0.05) and absolute log2 Fold Change (> 0.5). The enriched blood transcription modules were obtained by the Hypergeometric test. **(A)** Modules related to innate immunity and antigen presentation, **(B)** modules related to cell cycle, adaptive immunity and cytokines modules. The effect size is proportional to the size of the pie, while the adjusted p-value is proportional to colour intensity. Within each pie, the proportion of significantly upregulated and downregulated genes is shown in red and blue, respectively. The gray portion of the pie represents genes that are not significantly differentially regulated.

The enrichment analysis was performed using the *Blood Transcription Modules (BTM)* database and the *tmod* package, the complete results of the different groups are reported in the **Supplementary Data Sheet 1**. **Figure 5** shows a summary of the main immune system modules activated for each comparison and time point. In total, 87 modules were significantly enriched, only 3 of them being specifically activated in infected samples, while 40 modules were only activated after stimulation of previously infected samples.

#### 3.3.1 Activation of Extracellular Matrix, Cell Adhesion, and Innate Immune Response Modules

Five modules were consistently activated at almost all time points in both unstimulated and *in vitro* stimulated groups. Related to monocytes, immune activation, TLR signaling, and cell cycle, these modules showed a different pattern after stimulation, presenting more down-regulated genes. Following the same direction, modules related to the extracellular region, monocytes, and cell cycle are especially enriched in down-regulated genes after stimulation (**Figures 5A, B**).

The “extracellular region cluster” module shows the downregulation of genes involved in the interaction with extracellular components, growth control, and the vascular endothelium/angiogenesis (HSPG2, GH1, ENG). Moreover, the monocyte chemoattractant CCL2 is also down-regulated, while CCL18, important for the recruitment of T lymphocytes but not monocytes, is up-regulated.

The stimulation down-regulates genes responsible for the proliferation and differentiation of monocytes and macrophages like CSF2RA, CSF1R, and CSF3R, the latter one also important for adhesion. In monocytes modules, other genes linked to the extracellular matrix and cell adhesion followed the same behavior.

The downregulation of extracellular matrix genes could be due to the process of *in vitro* stimulation, decreasing the cell adhesion to the plate surface.

#### 3.3.2 Activation of Cell Cycle, Cytokines, and Adaptive Immune Response Modules

The stimulation of infected samples led to the enrichment of many biological pathways not activated in the previous comparisons, including modules related to antiviral response, antigen presentation, T cells, B cells, and chemokines (**Figures 5A, B**).

On the first day, unstimulated samples presented the enrichment of T cell and cell cycle modules. After stimulation, these same modules are activated, together with many others related to T cells and cell cycle, in both cases enriched mainly by down-regulated genes.

In T cell modules we find down-regulated cell-cycle genes and genes linked to cell adhesion, like VCAM1 and SIR3PG, while ITGA4, another adhesion-related gene, was up-regulated in unstimulated samples but presented no change after stimulation. Negative regulators of the T cell activity (LILRB4, LILRB3, SIT1) were also downregulated, while the few up-regulated genes were mainly related to T cell activation (CD3E, GRAP2, CDCA7, and LAT).

On the other hand, the specific modules in late time points were mostly activated by up-regulated genes. We observe a stronger activation of the “cytokines − receptors cluster” module and the specific enrichment of pathways like leukocyte differentiation, signaling in T cells, enriched in B cells, among other modules.

Cytokine modules are activated from the stimulation of baseline samples and looking inside these modules, indeed we see many up-regulated genes independently of the time point, especially those from the CCL family, IL1A, IL1RN, and TNF. Other genes such as CSF2, IL2RA, IL6, IL10 and IL1B present a modest increase in stimulation of baseline samples, but a major up-regulation at late time points.

Despite the high number of activated modules, the response to the stimuli after a previous infection does not show general up-regulation of the immune and inflammatory response. This second contact with the pathogen through the *in vitro* stimulation permitted us to appreciate biological processes which could not be detected in the primary infection, especially those related to antigen presentation, adaptive immunity, and cytokines. These processes are possibly related to a recall immune response starting within the first days after infection.

### 3.4 Cytokines Assay Suggests the Promotion of Innate and Adaptive Immune Responses From Day 4 After Infection

Regarding the concentration of cytokines in splenocyte culture supernatants, the infection without subsequent stimulation did not result in significant increases in the concentration of cytokines, with exception of IL-17a at day 7 after infection (data not shown).

**Figure 6:**
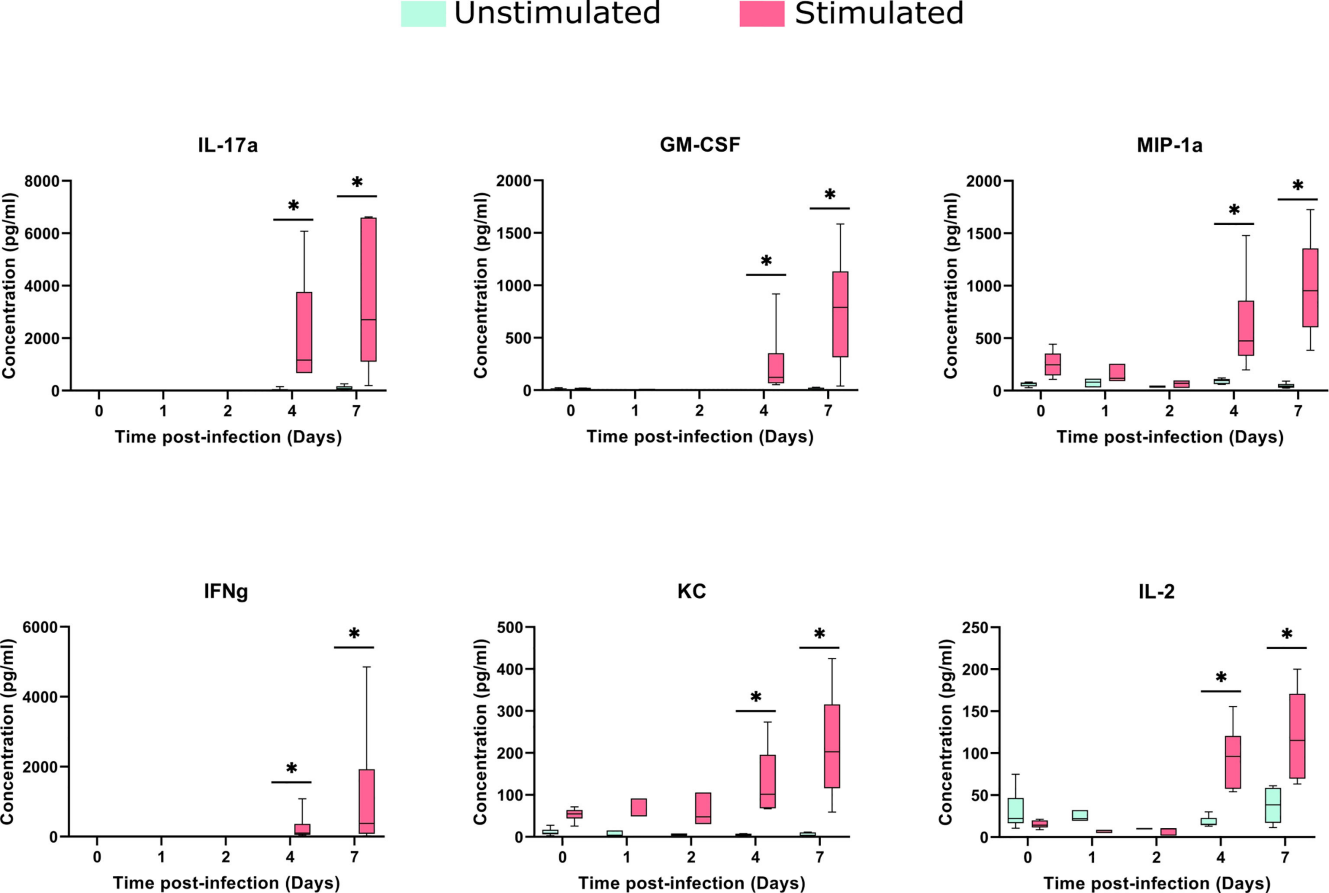
Cytokines concentrations in the spleens after TIGR4 pneumococcal infection. The spleens were collected from infected mice at different time points and splenocytes were cultured for 72 hours in the presence or not of a stimulus (formalin-inactivated TIGR4 pneumococcal strain). The supernatants were collected and the concentration of 23 cytokines were assessed by Luminex immunoassay. Data was analyzed using GraphPad Prism software. To compare Stimulated to Unstimulated samples we used the Wilcoxon paired test. Concentrations from six of the cytokines are presented in the figure (for all the 23 cytokines see **Supplementary Image 3**). When compared to only infected samples, all the six cytokines presented a significant (*= *P*<0.05) increase when stimulated in days 4 and 7 after infection.

The stimulation process induced a significant increase in KC and MIP-1a, compared to baseline samples (median of differences of 42.46 and 184.4 pg/ml, respectively). Despite cytokine changes between stimulated and control samples being noticeable in early stages, they increased considerably upon *in vitro* stimulation, at days 4 and 7 after the infection (**Figure 6**).

The comparison of stimulated samples from days 4 and 7 after infection with only infected samples showed a significant increase in all cytokine concentrations, with exception of MCP-1 and IL12p40 (**Figure 6** and **Supplementary Image 3**), suggesting the involvement of both innate and adaptive branches of the immune system. This increase was more accentuated on day 7, in which the difference in the median between the stimulated and unstimulated groups was 2597 pg/ml for IL-17A, 769 pg/ml for GM-CSF and 374 pg/ml for IFN-gamma.

### 3.5 Gene Expression and Cytokines Data Integration Indicate Specific Patterns of Recall Immune Response After Stimulation

To identify the genes correlated with the increase in the concentration of cytokines, especially at late time points, data integration was performed using the sparse version of Partial Least Squares (sPLS), from MixOmics package. PLS can integrate two types of data measured on the same sample by maximizing the covariance between the components of each data set. The sparse version applies LASSO ℓ1 penalizations in PLS analysis to perform feature selection.

**Figure 7:**
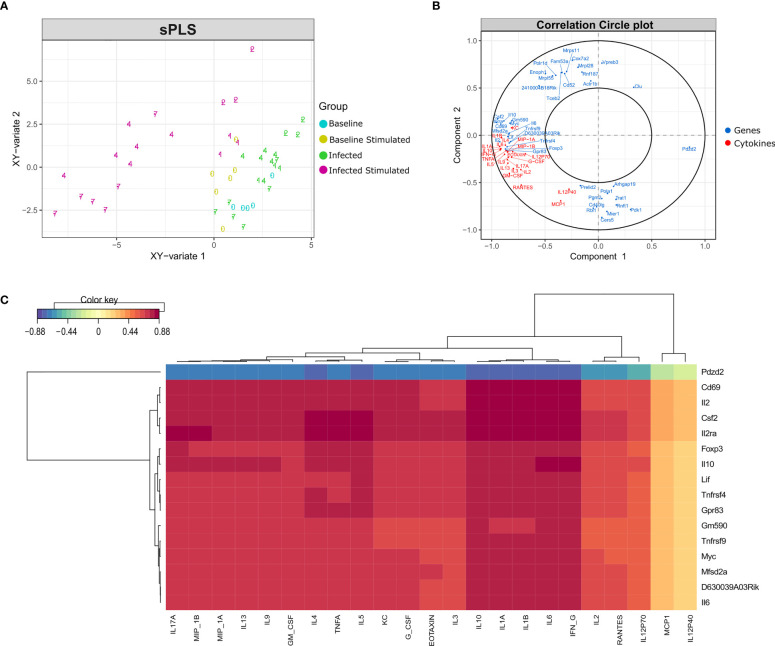
Data integration using sPLS (Sparse Partial Least Squares). Data integration suggests a different pattern in stimulated samples from days 4 and 7 after infection, driven by the expression of a few genes and the concentration of most cytokines. **(A)** sPLS: Partial Least Squares regression applying the tune.spls function (MixOmics), 16 genes were selected for component 1 and 25 genes for component 2. Colors represent samples from different groups: infected or non-infected and with or without *in vitro* stimulation. Numbers indicate the study days. **(B)** Correlation Circle Plot: the selected genes (blue) and cytokines (red) are represented in a correlation circle plot. Variables positively associated are projected in the same direction from the origin, variables negatively correlated are projected in opposite directions. Variables displayed in a perpendicular angle are not correlated and the greater the distance from the origin the stronger the association. For example, the cytokine IL1B is positively correlated with the IL2 gene, but is negatively correlated with the gene Pdzd2. In general, cytokines and several genes related to cytokines are accumulated on the left side of the graph, indicating a high correlation among them. **(C)** Clustered Image Map (CIM): correlation between genes (mRNA), reported in vertical, and Cytokine production, in horizontal. The map highlights the correlation values for these variables for the genes selected on the first component of the sPLS. High positive correlations are represented by dark red, while high negative correlations by dark blue. Genes related to cytokines such as Il2ra, Csf2, Il10, Il6 and Il2 are positively correlated with most cytokines concentration. The select genes are strongly correlated with almost all cytokines, with exception of MCP1 and IL12p40.

As expected, the *in vitro* stimulated samples formed a different cluster compared to the non-stimulated samples, although there is a different behavior regarding time points in each cluster (**Figure 7A**). In the non-stimulated cluster there is a perturbation caused by infection, but some samples from day 7 cluster together with control samples from day 0. On the other hand, *in vitro* stimulated samples presented a different pattern, samples from days 4 and 7 form a new cluster, driven by the increase in cytokine concentration and the expression of certain genes (**Figure 7B**).

By performing data integration and feature selection, sPLS identifies the genes whose expression is strongly associated with the concentrations of cytokines, providing the correlation value for each variable. The genes with the highest values of correlation with the 23 cytokines were Cd69, Csf2, Il2ra, and Il2 (**Figure 7C**). Other genes related to the immune system (Foxp3, Tnfrsf4, Tnfrsf9, Il10, and Il6) were also found positively correlated with the cytokines.

### 3.6 Possible Biomarkers Elicited by *In Vitro* Stimulation

We aimed to understand if feature selection could summarize the impact of a previous infection on stimulated samples, indicating possible biomarkers of this infection. We applied the DaMiR-seq package, which provides data normalization, feature selection, and classification, based on different machine learning techniques. Three groups were established based on the transcriptomics and cytokine data distribution, focusing on the stimulation of uninfected samples, samples from early time points after infection (days 1 and 2), and samples from late time points (days 4 and 7).

**Figure 8:**
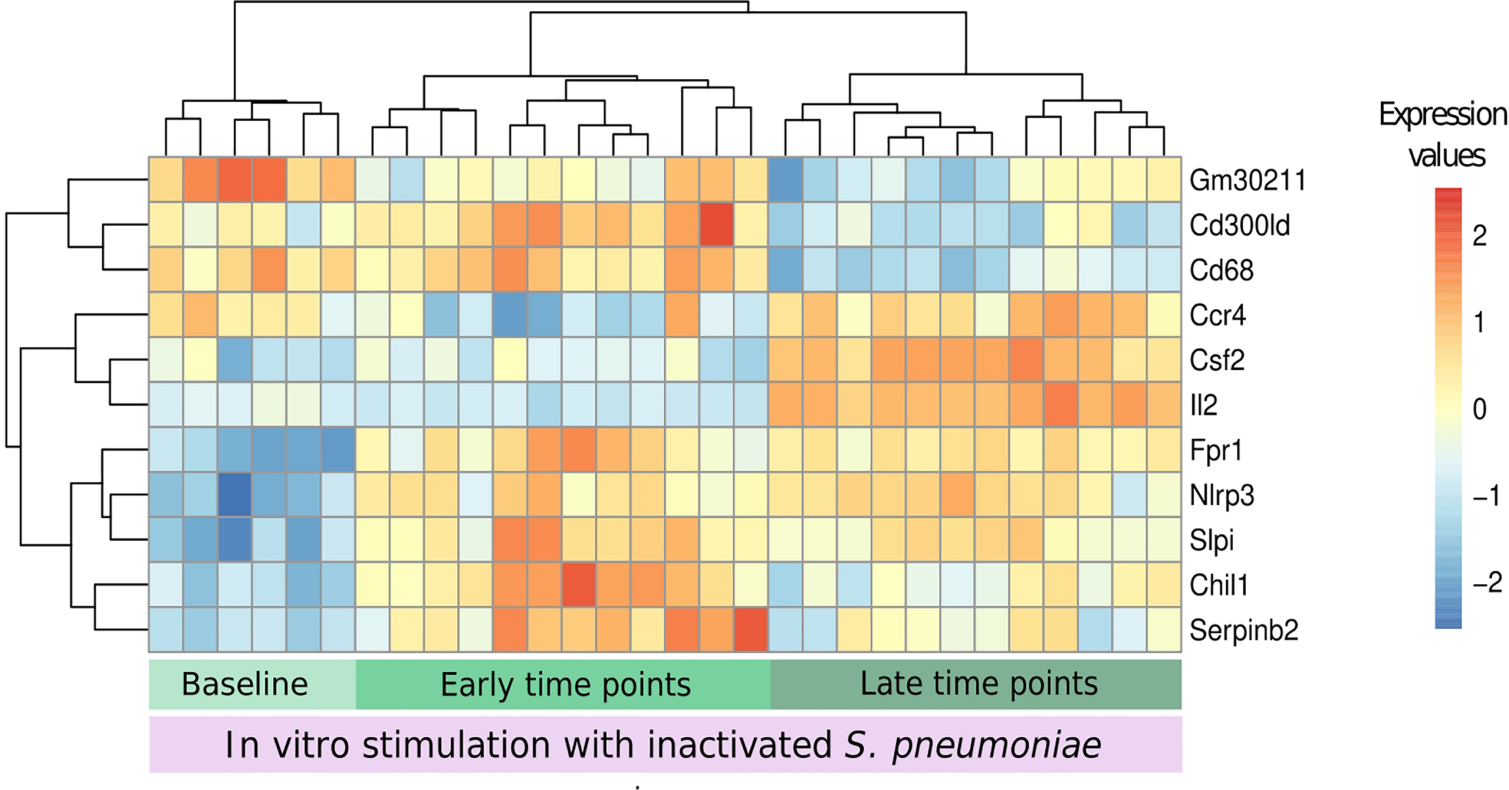
Biomarkers of recall immune response. The DaMiR-seq package combines different classification methods to select possible biomarkers. After selecting the stimulated samples, this package was used to find genes involved in the differences between infected and uninfected samples after stimulation. Groups were established based on the results of data distribution (IPCA and sIPCA) and cytokines, where we observed a shared pattern between early time points (days 1 and 2) and another pattern at late time points (4 and 7). Eleven genes were selected based on the drop of the feature importance (**Supplementary Image 1**). The normalized expression values of the selected genes permitted to form three clear clusters.

Eleven genes were chosen by applying a threshold of 0.5 to the scaled importance score identified by the DaMiR-seq package (**Supplementary Image 1**). These 11 genes allowed a clear clusterization of the three groups (**Figure 8**).

When compared to baseline stimulated samples, Fpr1, Nlrp3, and Slpi presented an increased expression in infected stimulated samples, independently of the time point. The stimulation of samples from early time points after the infection led to the increase in the expression of other inflammatory genes like Serpinb2 and Chil1 (Chitinase-3-like protein 1), and these values started to decrease in the subsequent days. At late time points, three other important genes, related to cytokine activity, had their expression increased when compared to the other groups: Ccr4, Csf2, and Il2.

Despite the small number of samples being a limitation for this type of analysis, the feature selection summarizes the new immunological processes that arise after the stimulation of infected samples and suggests the use of the *in vitro* stimulation model to detect the presence of a previous pneumococcal infection by measuring the expression of a few genes.

## 4 Discussion

To characterize the host response to *S. pneumoniae* we proposed a murine model of intranasal infection followed by an *in vitro* stimulation of splenocytes with inactivated bacterial whole cells, at different time points after infection. Using a transcriptomic-based approach, our study has highlighted genes and biological pathways associated with the stimulation of baseline and previously infected samples, as well as the cytokines involved in the same processes.

In accordance with the genes selected by the sIPCA, the enrichment analysis has shown that the simple presence of inactivated bacteria leads to the activation of cytokines genes and different immune system pathways, mainly related to innate immunity. However, when this stimulation occurs in previously infected samples, there is a higher number of DEGs, revealing 40 new biological modules distributed across time points.

Stimulated samples presented downregulation of monocytes modules, together with the upregulation of antigen presentation and cytokine modules, which were also reported in gene expression data from alveolar macrophages (AM) in a pneumococcal colonization study, when comparing volunteers that developed carriage or not after experimental human pneumococcal challenge (Mitsi et al., 2020). In fact, the study did not suggest an increase in monocytes, but a higher monocyte-AM differentiation in people that developed carriage. On the other hand, monocytes seem to be recruited at the nose after the establishment of carriage (Jochems et al., 2018).

Recent vaccine studies have emphasized that innate immunity modules, including antigen presentation and dendritic cell activation, demonstrate stronger activation after a second contact with the antigen. Using an *in vivo* boost with the antigen alone following the priming with a chimeric vaccine against *Mycobacterium tuberculosis*, Santoro et al. have observed a faster and more robust response of dendritic cells and antigen presentation (Santoro et al., 2018). Similar results were recently observed in a different context, with an mRNA vaccine against SARS-CoV-2, in which the second dose activated new antigen presentation modules (Arunachalam et al., 2021).

Transcriptomics results have shown the presence of a particular response after a second contact with the pathogen and the concentrations of the cytokines suggested that this response is marked by different patterns of activation, with the stimulation allowing a better classification between early time points (1 and 2 days) and late time points (4 and 7) after infection. Moreover, the activated modules and the concentration of the soluble modulators suggested that both innate and adaptive branches of the immune system are promoted by the stimulation, suggesting cooperation between them.

In our model, cytokines secreted by macrophages, like MIP-1a (CCL3), KC (CXCL1), IL-1a, IL-1b, and IL-6 had a small, although significant, increase after stimulation of baseline samples. After stimulation of early time points, a small increase in the concentration of MIP-1b, RANTES, and TNF-a was observed, although not statistically significant. When the *in vitro* stimulus occurs at late time points after infection, the concentration of all these cytokines significantly increases, especially at day 7. In fact, innate immune responses to pneumococcus are known for polarization towards Th1 and Th17 responses through the release of cytokines (Bogaert et al., 2009).

Following this reasoning, it was expected that after the stimulation of baseline samples the cytokines linked to the adaptive immunity activation such as IL-17, IFNg, and IL-2 did not present an increased concentration in comparison to unstimulated samples. G-CSF and GM-CSF presented a small increase, although not significant. Again, at early time points no important changes are seen, but at late time points, a significant increase in the concentration was observed for all these cytokines, suggesting the activation of a Th1 and Th17 response starting around day 4. A strong Th1 response characterized by high levels of IFNg was also demonstrated in a murine model of bacterial meningitis by type 4 *S. pneumoniae*, already 48 hours after infection. (Pettini et al., 2015).

A previous study has highlighted the action of CD8+ T cells in helping AM to develop high MHC II expression after adenovirus infection, a process that started coincidently with the entry of T cells in the alveolar tissue, around 5 days after the infection (Yao et al., 2018). The activation of T cells in the spleen could follow a similar behavior in supporting macrophage activity and consequently increasing cytokine release.

Biomarker analysis and sPLS integration were employed to find genes that characterize the recall immune response and correlate with the increase in the concentration of the cytokines. Among the genes found positively correlated by the sPLS method, many were linked to the immune response. The TNF receptors Tnfrsf9 and Tnfrsf4, are important for Th1 promotion and CD4 responses and, together with Il2, Il2ra, Foxp3 and IL10 participate in the “NF-kappaB signaling” biological pathway (Cho et al., 2021). Moreover, these genes are also associated with regulatory T cells, along with Cd69 and Il6, two other features found correlated with cytokines in the same analysis (Maloy and Powrie, 2005; Kimura and Kishimoto, 2010; Chaudhry et al., 2011; Yu et al., 2018; Hinterbrandner et al., 2021).

The eleven genes selected as possible biomarkers are capable of correctly clustering the stimulated samples in the studied groups (baseline, early, and late time points, **Figure 8**). These genes could be cross validated in future studies using the same model of pneumococcal lung infection to study vaccine strategies and antimicrobial therapies. A link with pneumococcal infection, colonization, or vaccination was established in the literature for most of the selected genes. The Il2 and Csf2 genes were not only the first and third most important genes for the classification of samples regarding the presence of a previous infection but they were also among the genes with the highest correlation with the concentration of different cytokines, together with the Il2ra gene. This highlights the importance of the IL-2 signaling pathway to the described recall response. Indeed, different vaccine studies reported an increase in IL-2 cytokine after restimulation with pneumococcal proteins or peptides from these proteins (Kataoka et al., 2011; Singh et al., 2014; Elhaik Goldman et al., 2016; Converso et al., 2017).

Csf2 gene encodes for Granulocyte/Macrophage colony-stimulating factor (GM-CSF), a cytokine that presented one of the highest concentrations after stimulation of infected samples from late time points. Previous studies have described the increase in Csf2 expression and GM-CSF concentration in the lungs from mice infected intranasally with *S. pneumoniae* (Steinwede et al., 2011). *In vitro* stimulation of PBMCs with *S. pneumoniae* has also increased the concentration of this cytokine. Furthermore, a protective role of GM-CSF in pneumococcal infection was described with intra-alveolar administration of this cytokine (Schmeck et al., 2004; Steinwede et al., 2011) and the resistance to lung infection attributed to the microbiota was found to be through GM-CSF signaling (Brown et al., 2017).

The lack of Fpr1 and Chil1 led to a higher mortality rate in murine models of pneumococcal meningitis and pneumonia, respectively (Dela Cruz et al., 2012; Oldekamp et al., 2014).

Slpi is involved in the innate immune response to bacterial infections, regulating the NF-kappa-B activation and inflammatory responses. This gene was up-regulated in the lungs of mice infected with pneumococcus, but the same was not observed in the spleen, suggesting that its expression is modulated at the site of inflammation in the presence of inflammatory stimuli (Abe et al., 1997). Our data suggested a similar result, since Slpi expression did not change in the spleen of infected samples, but only increased after the *in vitro* stimulation.

Cd300ld presents no clear link with pneumococcal infection, but its encoded protein, an activating receptor in myeloid and mast cells, was downregulated in the blood of mice infected with *Streptococcus suis* (Dai et al., 2018).

The link of most of the selected genes with the physiopathology of pneumococcal infection supports the use of feature selection and machine learning techniques to unveil gene signatures, potentially finding new features and/or assigning new roles to genes involved in a process, such as recall responses. The changes in cytokines concentration and gene expression are two important ways to assess immunological information after infection or vaccination. Our findings suggest that *in vitro* stimulation is an important step to understanding the systemic response to pneumococcal lung infection and the immunological memory generated by this bacteria. The analysis of transcriptomic and cytokine data revealed a clustering of the samples based on the stage of infection (early vs late), with more intense signals at late time points. Integrative analysis identified few genes, related to the immune system, which could categorize the samples based on the infection stage and which may be useful in future studies to monitor vaccine immune response and experimental therapies efficacy.

## Data Availability Statement

The datasets generated for this study can be found in the GEO database under accession number: https://www.ncbi.nlm.nih.gov/geo/query/acc.cgi?&acc=GSE199605. The bioinformatic analysis can be accessed at https://github.com/IsaMoscardini/Spleen_stimulation.

## Ethics Statement

The animal study was reviewed and approved by the Italian Ministry of Health with authorization n° 304/2018-PR.

## Author Contributions

GP, FI and FS conceived and designed the experiments. FS and MC prepared the bacteria for animal infection and for *in vitro* stimulation. MC, FF, SG and EP performed animal experiments. MC and CG performed transcriptomic analysis. MC and FF analysed cytokines. DM secured funding. IM analysed data and drafted the paper with contributions from FS, AG and GP. All the authors reviewed, edited and approved the final version of the manuscript.

## Funding

This study was carried out with financial support from the Commission of the European Communities, Seventh Framework Programme, Innovative Medicines Initiative Joint Undertaking “Biomarkers for Enhanced Vaccine Safety” project BioVacSafe (IMI JU Grant No. 115308).

IM received a PhD fellowship under the Marie Sklodowska-Curie actions (MSCA) – Innovative Training Networks (ITN), Project VacPath (Novel vaccine vectors to resist pathogen challenge) grant agreement No 812915 funded by the European Union’s Horizon 2020 research and innovation programme.

## Conflict of Interest

Authors IM and AG are employed by Microbiotec srl.

The remaining authors declare that the research was conducted in the absence of any commercial or financial relationships that could be construed as a potential conflict of interest.

## Publisher’s Note

All claims expressed in this article are solely those of the authors and do not necessarily represent those of their affiliated organizations, or those of the publisher, the editors and the reviewers. Any product that may be evaluated in this article, or claim that may be made by its manufacturer, is not guaranteed or endorsed by the publisher.

## Supplementary Material

Supplementary Image 1 Feature Importance. The DaMiRseq package ranks the selected features using RReliefF, a multivariate filter technique that assesses the relevance of the features. The graph shows the importance of each feature, indicating that Il2 gene is the best classificator.

Supplementary Image 2 Independent Principal Component Analysis (IPCA). The IPCA analysis from mixOmics package displays the distribution of samples by their gene expression, pointing out two main clusters that split samples according to their stimulation status. Numbers represent the time point of each sample, leading to the formation of three groups: baseline (light green), early time points - days 1 and 2 (green), and late time points - days 4 and 7 (dark green). The purple shading highlights the clusters composed by stimulated samples.

Supplementary Image 3 Cytokines boxplots (complete panel). Boxplots comparing the concentration of 23 different cytokines in stimulated and unstimulated samples at baseline and at different time points after infection.

Supplementary Data Sheet 1 Modules enriched in each comparison and time point.
