# Supplementary material for: Immune Memory After Respiratory Infection With Streptococcus pneumoniae Is Revealed by in vitro Stimulation of Murine Splenocytes With Inactivated Pneumococcal Whole Cells: Evidence of Early Recall Responses by Transcriptomic Analysis
Source: Front Cell Infect Microbiol. 2022 Jun 20;12:869763. doi: 10.3389/fcimb.2022.869763 (PMC9251119; doi:10.3389/fcimb.2022.869763)
Supplement: Supplementary Image 1 — Feature Importance. The DaMiRseq package ranks the selected features using RReliefF, a multivariate filter technique that assesses the relevance of the features. The graph shows the importance of each feature, indicating that Il2 gene is the best classificator. [file Image_1.pdf]

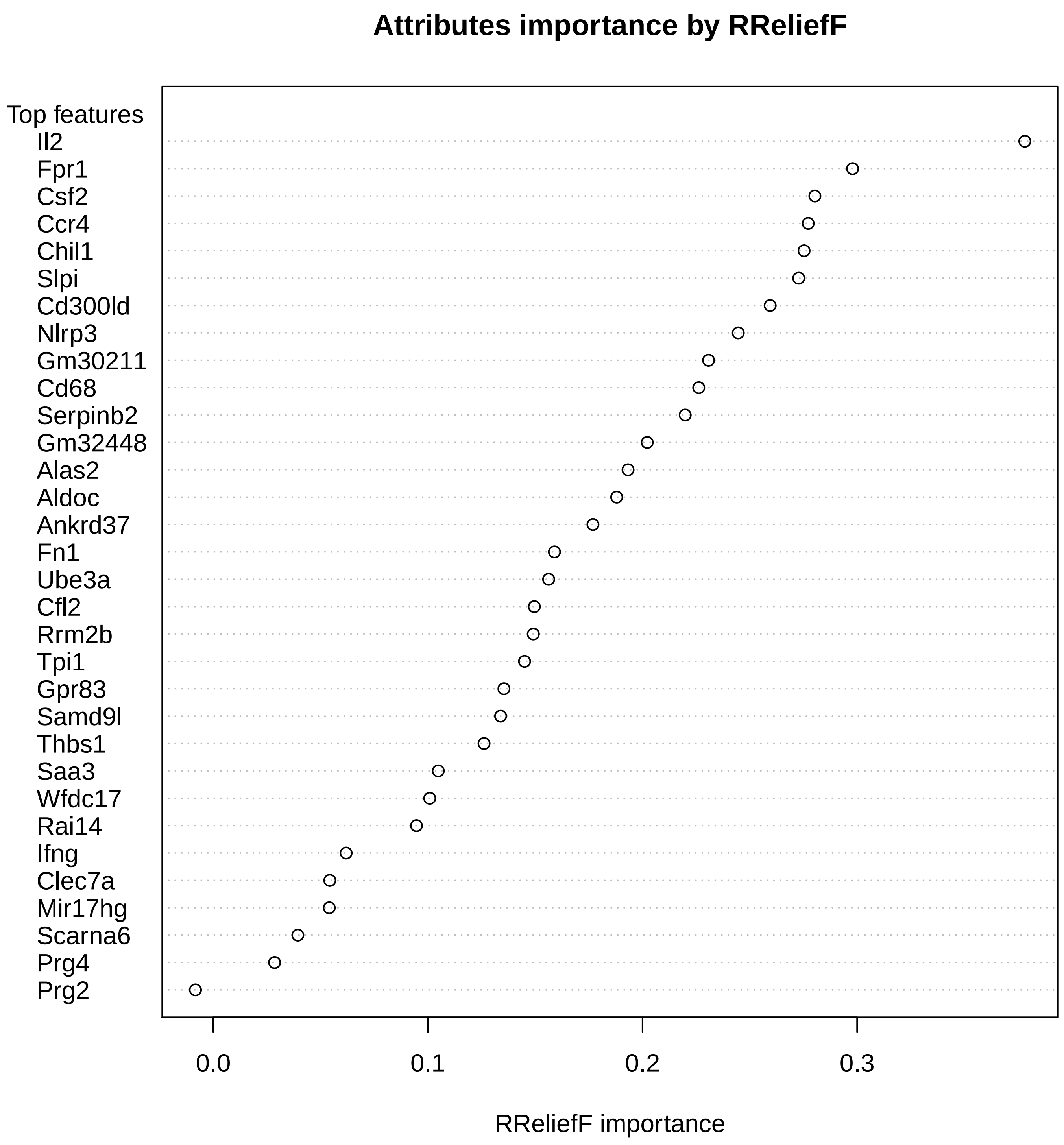
Attributes importance by RReliefF
Top features
Il2
Fpr1
Csf2
Ccr4
Chil1
Slpi
Cd300ld
Nlrp3
Gm30211
Cd68
Serpinb2
Gm32448
Alas2
Aldoc
Ankrd37
Fn1
Ube3a
Cfl2
Rrm2b
Tpi1
Gpr83
Samd9l
Thbs1
Saa3
Wfdc17
Rai14
Ifng
Clec7a
Mir17hg
Scarna6
Prg4
Prg2
0.0
0.1
0.2
0.3
RReliefF importance
