# Supplementary figures and images for: Immune Memory After Respiratory Infection With Streptococcus pneumoniae Is Revealed by in vitro Stimulation of Murine Splenocytes With Inactivated Pneumococcal Whole Cells: Evidence of Early Recall Responses by Transcriptomic Analysis
Source: Front Cell Infect Microbiol. 2022 Jun 20;12:869763. doi: 10.3389/fcimb.2022.869763 (PMC9251119; doi:10.3389/fcimb.2022.869763)

# IPCA

PC2: 12% expl. var

0.2

0.0

-0.2

-0.4

-0.2

-0.1

0.0

0.1

0.2

PC1: 10% expl. var

- Baseline
- Early time points
- Late time points
- Stimulated samples

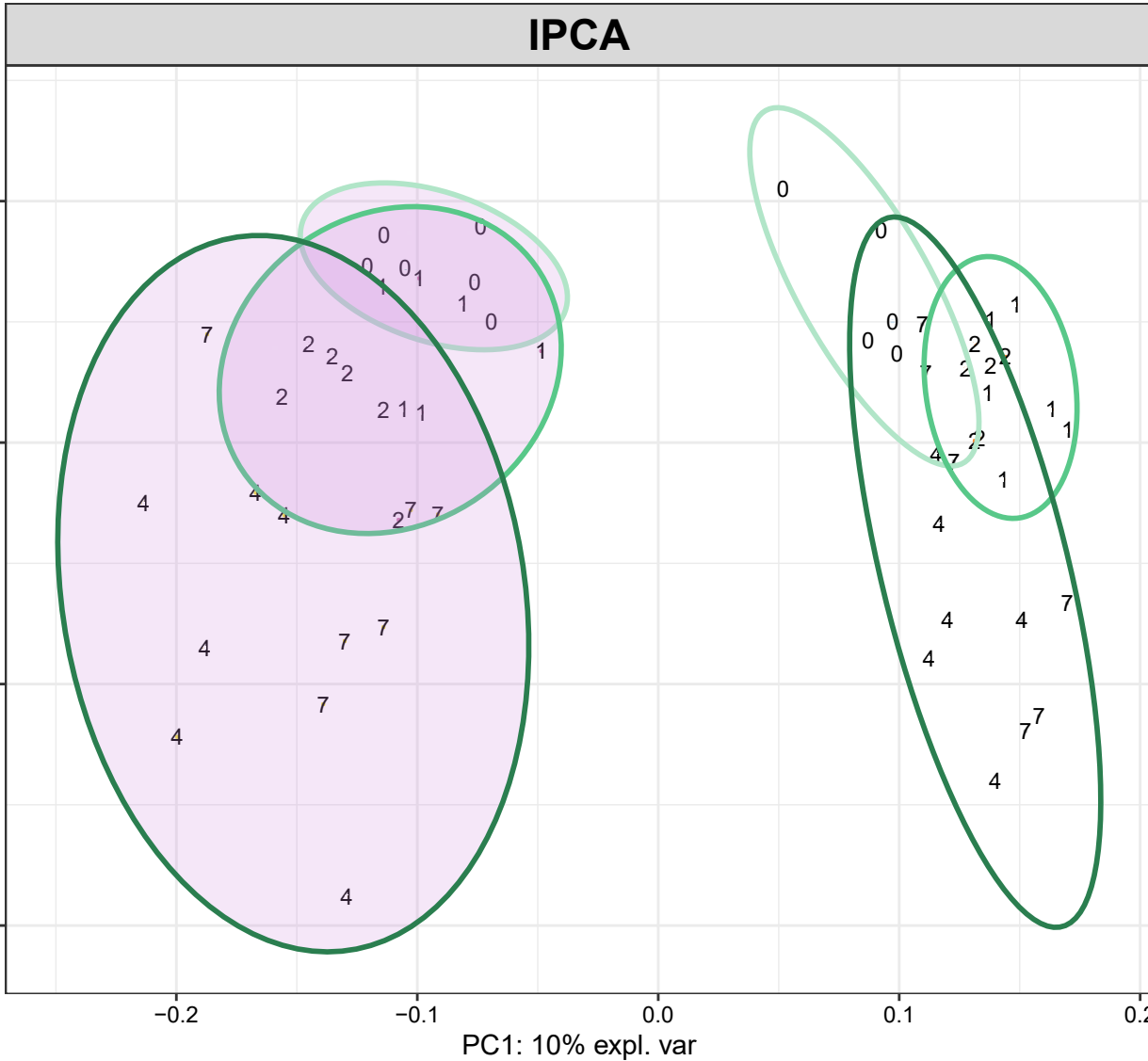

Supplement: Supplementary Image 2 — Independent Principal Component Analysis (IPCA). The IPCA analysis from mixOmics package displays the distribution of samples by their gene expression, pointing out two main clusters that split samples according to their stimulation status. Numbers represent the time point of each sample, leading to the formation of three groups: baseline (light green), early time points - days 1 and 2 (green), and late time points - days 4 and 7 (dark green). The purple shading highlights the clusters composed by stimulated samples. [file Image_2.pdf]

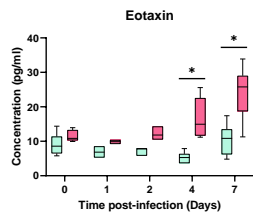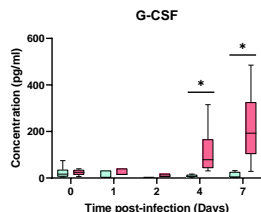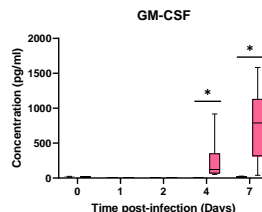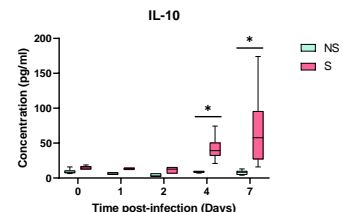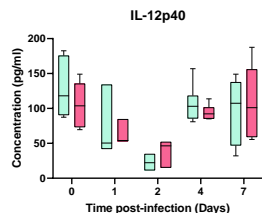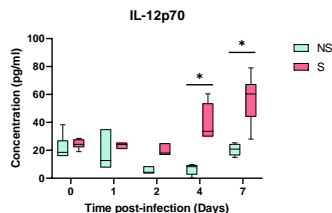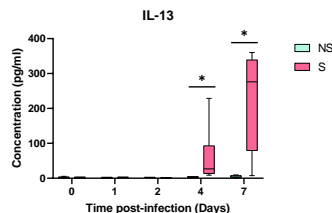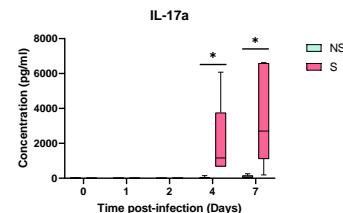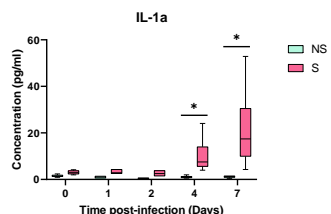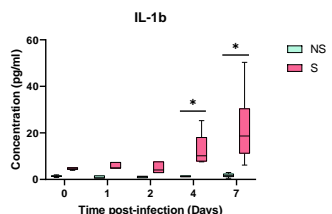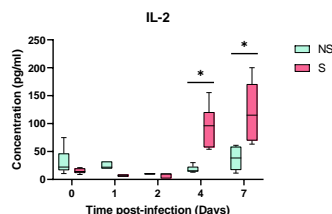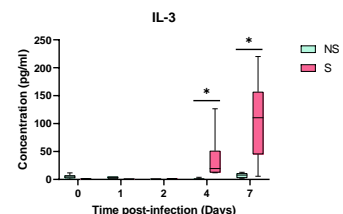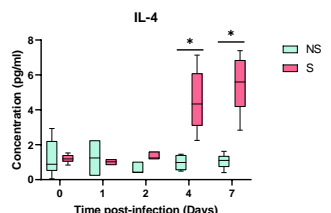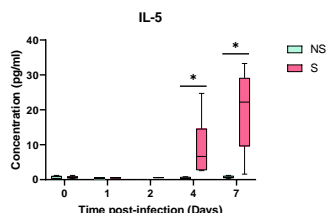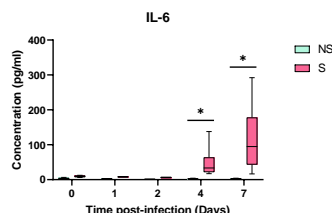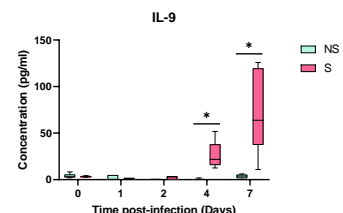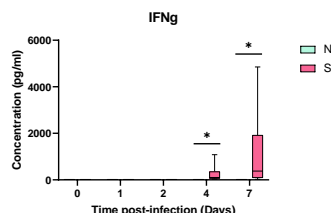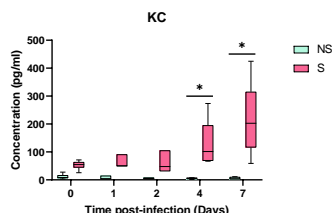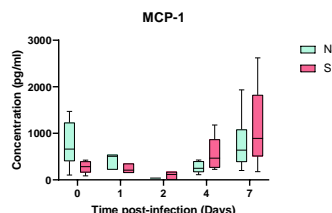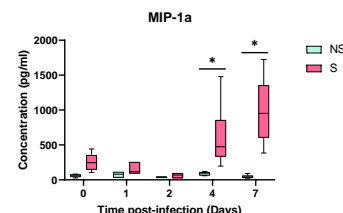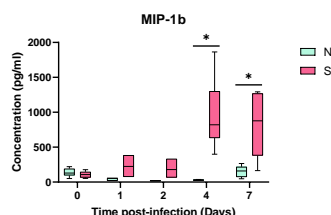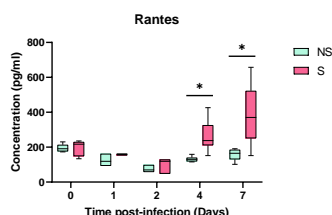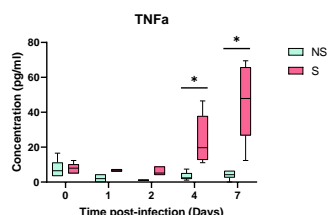

Supplement: Supplementary Image 3 — Cytokines boxplots (complete panel). Boxplots comparing the concentration of 23 different cytokines in stimulated and unstimulated samples at baseline and at different time points after infection. [file Image_3.pdf]
